# Hybrid Nanocellulose-Copper (II) Oxide as Engine Oil Additives for Tribological Behavior Improvement

**DOI:** 10.3390/molecules25132975

**Published:** 2020-06-28

**Authors:** Sakinah Hisham, Kumaran Kadirgama, Hussein A. Mohammed, Amit Kumar, Devarajan Ramasamy, Mahendran Samykano, Saidur Rahman

**Affiliations:** 1 Faculty of Engineering Technology Mechanical and Automotive, Universiti Malaysia Pahang, Pekan 26600, Pahang, Malaysia; sakinah.hisham0704@gmail.com (S.H.); kumaran@ump.edu.my (K.K.); deva@ump.edu.my (D.R.); 2 School of Engineering, Edith Cowan University, 270 Joondalup Drive, Joondalup, WA 6027, Australia; 3 College of Engineering, Mechanical Department, Universiti Malaysia Pahang, Pekan 26600, Pahang, Malaysia; mahendran@ump.edu.my; 4 Research Center for Nano-Materials and Energy Technology (RCNMET), School of Science and Technology, Sunway University, Bandar Sunway, Petaling Jaya 47500, Selangor, Malaysia; saidur@sunway.edu.my; 5 Department of Engineering, Lancaster University, Lancaster LA1 4YW, UK

**Keywords:** cellulose nanocrystal, copper (II) oxide, friction, wear

## Abstract

Friction and wear are the main factors in the failure of the piston in automobile engines. The objective of this work was to improve the tribological behaviour and lubricant properties using hybrid Cellulose Nanocrystal (CNC) and Copper (II) oxide nanoparticles blended with SAE 40 as a base fluid. The two-step method was used in the hybrid nanofluid preparation. Three different concentrations were prepared in a range of 0.1% to 0.5%. Kinematic viscosity and viscosity index were also identified. The friction and wear behavior were evaluated using a tribometer based on ASTM G181. The CNC-CuO nano lubricant shows a significant improvement in term of viscosity index by 44.3–47.12% while for friction, the coefficient of friction (COF) decreases by 1.5%, respectively, during high and low-speed loads (boundary regime), and 30.95% during a high-speed, and low load (mixed regime). The wear morphologies results also show that a smoother surface was obtained after using CNC-CuO nano lubricant compared to SAE 40.

## 1. Introduction

A suitable lubricant is primarily associated with its formulation, which means that the additives that contain in the lubricants play a vital role in enhancing its performance regarding reducing friction and wear. The lubricant with a suitable and relevant combination of base oil and additive helps in reducing energy loss in a mechanical system state by [1]. The use of the solid additive in lubricant not only reduces the friction coefficient but also increases the load capacity [2].

The applications of nanoparticles as a lubricant additive have steadily increased in recent years, as many researchers [3] demonstrated a reduction in the friction and wear of nanoparticle-containing lubricant formulation, which is also known as nano lubricant [4]. Researchers have developed a variety of additives to overcome and decrease tribological challenges such as wear, friction, oxidation, corrosion and the scuffing mechanism on the lubricant base stocks and to enhance the lubricant efficiency [4,5,6,7,8]. The main advantages of using nano lubricants are that they are relatively insensitive to temperature, and tribochemical reactions are limited compared to the traditional additives [9]. Various types of nanoparticles can be used, such as a polymer, metal, organic and inorganic materials like aluminium oxide (Al_2_O_3_), titanium oxide (TiO_2_), copper oxide (CuO), and multi-wall carbon nanotube (MWCNT). A smaller sized nanoparticle will improve the tribological behaviour and render its shape nearly spherical, exhibiting superior rolling, lower affinity to the metal surface and decreasing the contact temperature.

Due to the various advantages of nano lubricant, organic-inorganic, in other words hybrid nanoparticles, have attracted much interest due to their current and potential applications as they can combine useful chemical, optical and mechanical characteristics. The hybrid nanoparticle is a composition of two or more nanoparticles synthesised and dispersed in a base lubricant [10] to improve the properties of single materials due to its excellent enhancement in rheological properties [11]. In recent years, the dispersion of organic-inorganic nanoparticles such as Multi-Walled Carbon Nanotube (MWCNT) hybrid with various inorganic nanoparticles such as alumina and silica for tribological properties has been getting researchers’ and academicians’ attention as they contribute to friction and wear reduction [12,13]. There are a few research articles on Cellulose Nanocrystal (CNC) hybrid with another nanoparticle in the oil base liquid. CNC is known as non-toxic and biodegradable as it is plant based [14,15,16].

Friction in the mechanical system will not occur with the presence of lubricants, and the main factors of mechanical system failure are energy loss and lower efficiency. Therefore, lubrication is one of the most effective ways to reduce friction, lower additional heat, and also to help prevent energy loss and lower efficiency in industrial components and tools by lowering friction among the mechanical parts, which is the most critical property [17]. A good lubricant needs to be developed to reduce the wear and friction. The use of mineral oils as a lubricant has become a growing concern worldwide, especially to those who are interested in preventing environmental issues. This paper investigated a new process for the development of a lubricant that can give advantages to people because it may lower the energy cost (cost saving), reduce waste, and have positive impacts on the environment. Nanolubricants were selected because they have advantages such as the improvement of tribological properties and increment in thermal conductivity [18]. The purpose of the nano lubricant is to improvise natural wear and friction. Nanoparticles’ concentration will affect wear and friction when added to the base oil. However, a limitation of the concentration needs to be set because some lubricants already contain some additives.

In order to gain the benefit of additive in lubricant for tribological behaviour, further research is required, especially on the concentration of additive used and the parameters involved. The concentration of additive is believed to help friction and wear during the experiment. In the present research, the hybrid nanocellulose-copper (II) oxide was added into SAE 40 to study the characterization of hybrid CNC-CuO, the stability of the nanolubricant, viscosity of lubricant and the tribological behaviour of the nanolubricant.

## 2. Methodology

### 2.1. Nanoparticle Preparation and Characterization

The CNC used in this research was extracted from the acetate grade dissolving pulp from the Western Hemlock plant to be in white to slightly off-white gel form. CNC was purchased from Blue Goose Biorefineries Inc with a 7.4% of CNC *w*/*w* suspension. According to the manufacturer, CNC from Blue Goose Biorefineries Inc, it does not contain sulfate half ester moiety, which is a bioactive ingredient that can rust the metal; thus, the CNC was suitable to improve the friction and wear performance of an engine. Copper (II) Oxide (CuO) was procured from US Research Nanomaterials, Inc. (USA). Copper oxide nanoparticles appear as a brownish-black powder. The two-step method suggested by [2,5,9,19,20] was used in the preparation of nano lubricant samples with volume concentrations from 0.1% to 0.5%. There are two processes in this method, which are (i) synthesis of the nanoparticles into the powder form, and (ii) dispersion of the nanoparticles into the base fluids (SAE 40) to form a stable and homogeneous solution. Since CNC is in gel form, spray drying was proposed as a technically appropriate process to convert it into powder form [21]. For the preparation of CNC in powder form, the suspensions were spray dried with a mini blower. The moisture in these suspensions is quickly evaporated upon direct contact with the hot air flow through the orifice of the nozzle on the spray dryer, resulting in drying-out and stable CNCs flake form. Then, the flakes were pulverized into powder form. The CNC was then dry-mixed together with CuO. Nano lubricant samples with a solid volume fraction of 0.1%, 0.3% and 0.5% were prepared by adding CNC and CuO in SAE 40 by using a magnetic stirrer and an ultrasonic bath.

The characterization of CNC-CuO nanoparticles with suspension was carried out by Field Emission Scanning Electron Microscopy (FESEM) equipped with Energy Dispersive X-ray (EDX) from Jeol Japan and model number JSM-7800F was used. This device provides images at the very high magnification and resolution of 1.3 nm at 30 kV. EDX in this device can detect element identification and element surface mapping from sodium to Uranium. Transmission Electron Microscopy (TEM) from Jeol Japan model number JEM-2100 was used to identify the characterization of the CNC-CuO nanoparticle. The device integrates X-ray spectrometer which chemically characterizes the samples. The resolutions of the device were designed with 0.34 nm (point) and 0.20 nm (line) and the magnification power ranging from X 35 to X 750,000. The sample testing was prepared by dropping one drop of nano lubricant onto the carbon grid, which was cleaned with 100% ethanol beforehand. After the drop had dried in natural air for 15 min, the solid nanoparticle was obtained and undertaken for the imaging process.

### 2.2. Nanolubricant Preparation and Its Stability

Nanolubricant samples with a substantial volume fraction of 0.1%, 0.3% and 0.5% were prepared by adding CNC and CuO in SAE40 by using a magnetic stirrer and ultrasonic bath. Nanoparticles in suspension tend to agglomerate due to their high surface area and surface activity [20]. In this work, the evaluation of the stability of the nano lubricants was carried out using the sedimentation method and a UV-Vis spectrophotometer. The UV-Vis spectrophotometer from Pelkin Elmer with model number TGA 4000 was used in this experiment. The wavelength range for this UV-Vis spectrophotometer is 190 to 3300 nm. The device was operated at a constant wavelength of 1200 nm for each of nano lubricant sample. A transparent macro quartz cuvette with 2 mL volume was used to place all the concentration samples of the nano lubricant test inside the slots. The UV-Vis spectrophotometer was used to measure the attenuation beam of light after it passes through a sample or after a reflection from a sample surface. The absorption and the scattering of light was measured by comparing the light intensity of CNC-CuO nano lubricant with SAE 40 as the base fluid.

### 2.3. Kinematic Viscosity and Viscosity Index (VI)

In order to get the kinematic viscosity data, testing was done according to the American Standard Testing Method (ASTM) D445 coupled with a temperature-controlled bath of Cannon Instrument Company, United States of America, Model CT-500 Series II using a Cannon-Fenske Routine Model glass capillary viscometer with a 2 mm inner diameter from Cannon Instrument Company, United States of America. Thermal oil was used in order to get a stable temperature distribution inside the capillary tube in the range from 40 °C to 100 °C at each concentration and they were measured accordingly.

### 2.4. Tribological Testing

**Figure 1:**
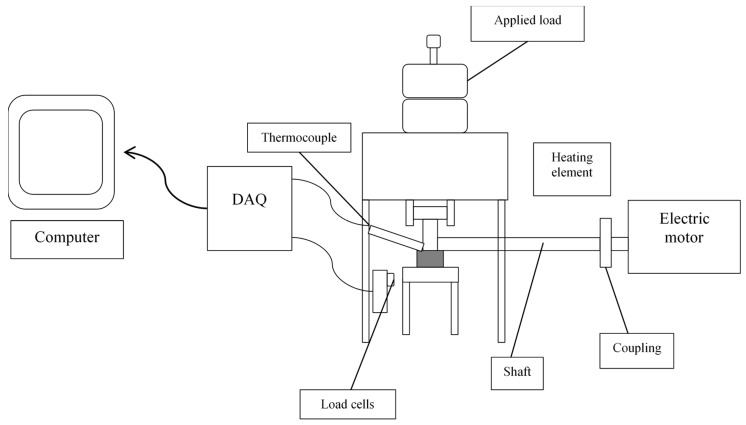
Schematic diagram of tribological testing.

The test was conducted using a custom-made friction and wear tester which also replicates the contact geometry relevant to the tribological phenomena occurring during the piston ring–cylinder liner contact in an engine. The schematic diagram of the friction pairs is shown in Figure 1. The wear test involves making linear reciprocates movements similar to a cylinder-piston ring pair operating under real conditions. A wear morphology that was caused at the surface of specimen and during the linear reciprocating sliding motion against the outer surface of aluminum 6061 for 30 min at the boundary lubrication regime (low speed and high load) in the presence of SAE 40 and different concentrations of nanoparticle (0.1%, 0.3% and 0.5%) added in SAE 40 was also reported. The temperature was 85 °C which is the regime temperature of the internal combustion engine and the operating time was 30 min per specimen. The coefficient of friction was recorded automatically using NI-DAQ via the ratio of friction force to normal load.

## 3. Results and Discussion

The main results show the CNC characteristics and the performance evaluation which was obtained by the analysis. The characterization was done by the thermo-physical observation of morphology, stability, and viscosity. The performance, meanwhile, was identified from the friction coefficient and wear mechanisms.

### 3.1. Nanoparticle Characterization

**Figure 2:**
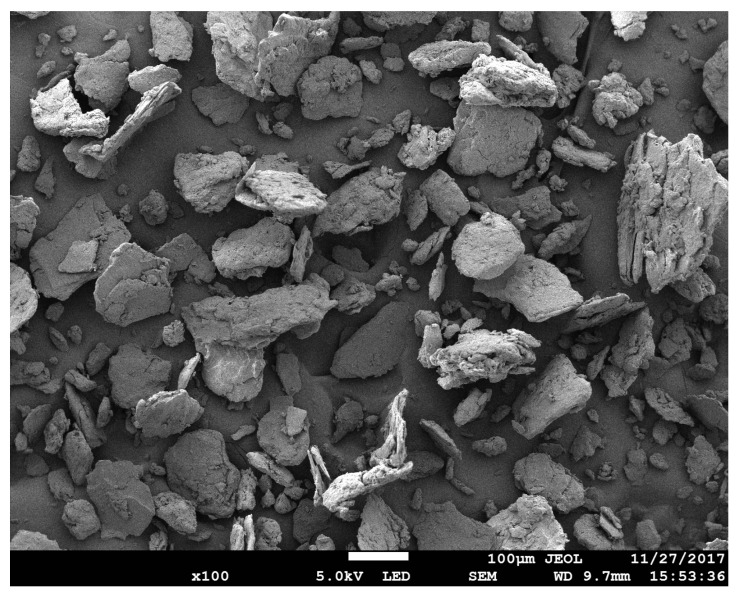
FESEM image for dry CNC.

**Figure 3:**
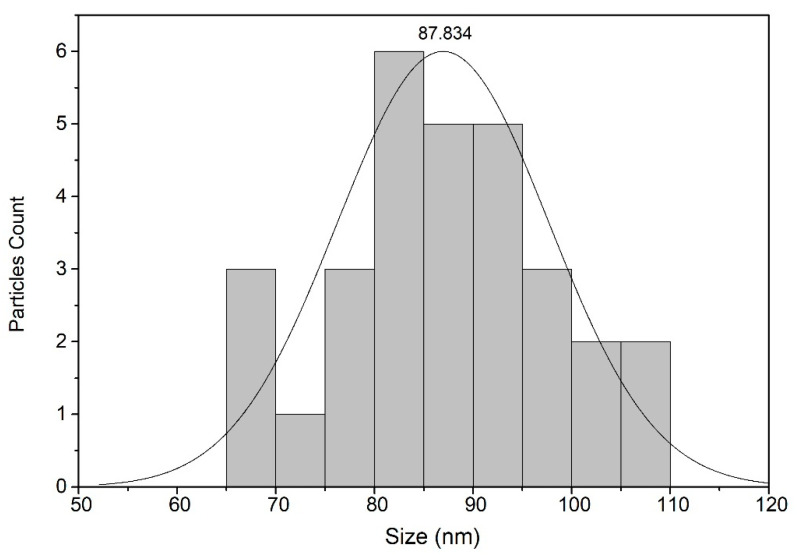
Particle size distribution.

The dry CNC under FESEM pictures are shown in Figure 2. The CNC images clearly shows a non-uniform size distribution. The particles are also shown to be spherical in shape and the average particle size is 82.6 nm, as shown in the histogram in Figure 3. Since the CNC gel water was dried with a hot air blower, CNC was agglomerated due to formation of irreversible hydrogen bonds between nanocellulose, which affect the nano scale size of nanocellulose so the independent CNC particles are not visible [22,23]. Table 1 shows the dimension information of CNC provided by the manufacturer.

**Figure 4:**
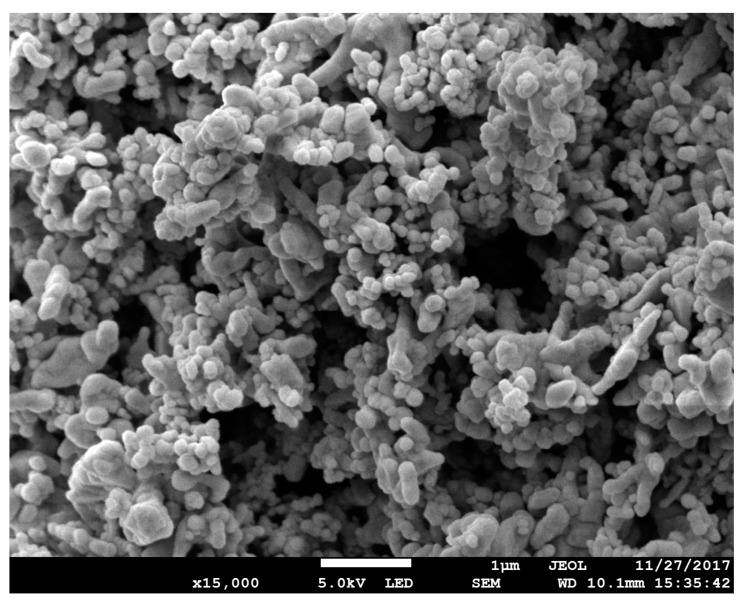
FESEM image for dry CNC-CuO nanoparticle powder.

**Figure 5:**
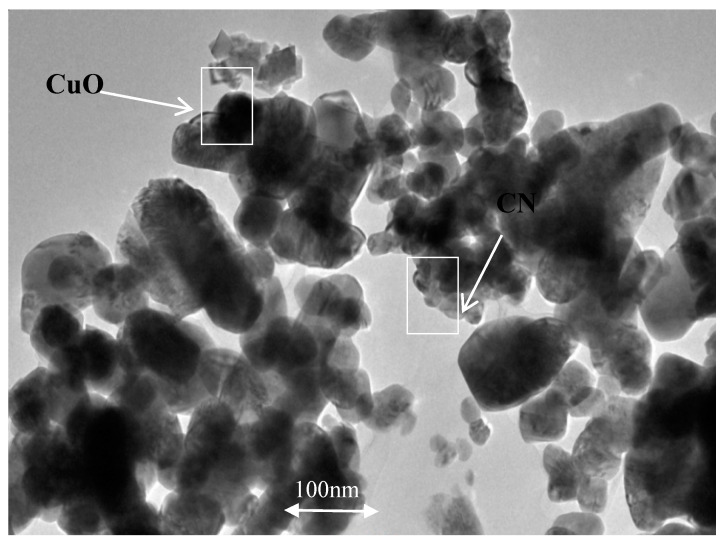
TEM image for CNC-CuO nanoparticle suspension.

**Figure 6:**
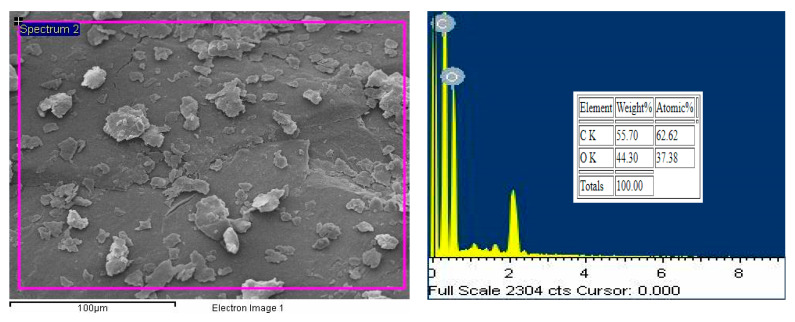
Area spectrum for CNC nanoparticles and EDX percentage of element in CNC nanoparticle.

**Figure 7:**
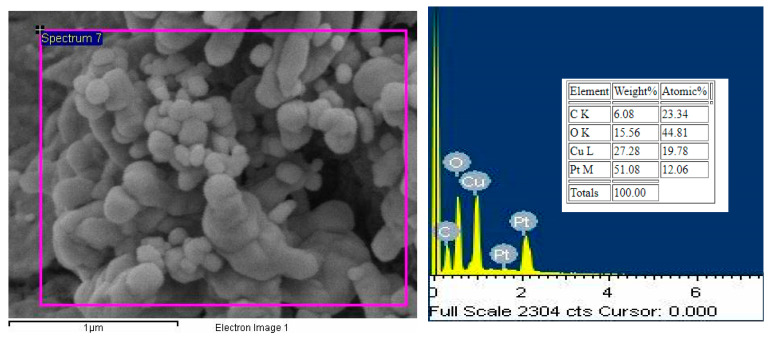
Area spectrum for CNC nanoparticles and EDX percentage of element in CNC nanoparticle.

The CNC picture at first also shows that the particles have the form of agglomerates, and it starts to separate into a more size-uniform particle after CuO is added, as shown in Figure 4. Agglomerates form the nanoparticle have to be broken by a magnetic stirrer and ultrasonic agitation to produce a stable nano lubricant [22,24]. This step was very important to ensure that the CNC-CuO nanoparticle were evenly distributed through the friction and wear specimen. Figure 5 shows the TEM pictures CNC-CuO nanoparticle suspension. TEM images also show that the suspension is homogeneously well dispersed, as shown in Figure 5. Figure 6 shows the EDX results for CNC nanoparticles. The EDX results show that only two elements were found at CNC, C (carbon) and O (oxygen), with a weight percentage of 55.70% for C and 44.30% for O. Figure 7 shows the EDX results for CNC-CuO and three elements were found in CNC-CuO, C with a weight percentage of 6.08%, O with a weight percentage of 15.56% and Cu with the highest weight percentage, 51.08%.

### 3.2. Suspension Stability of CNC-CuO in SAE 40

**Figure 8:**
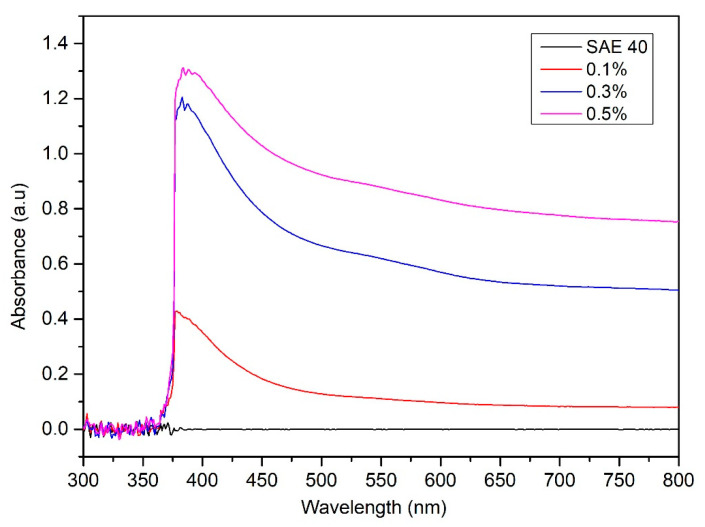
UV-Vis spectrum for a concentration from 0.1% to 0.5%.

**Figure 9:**
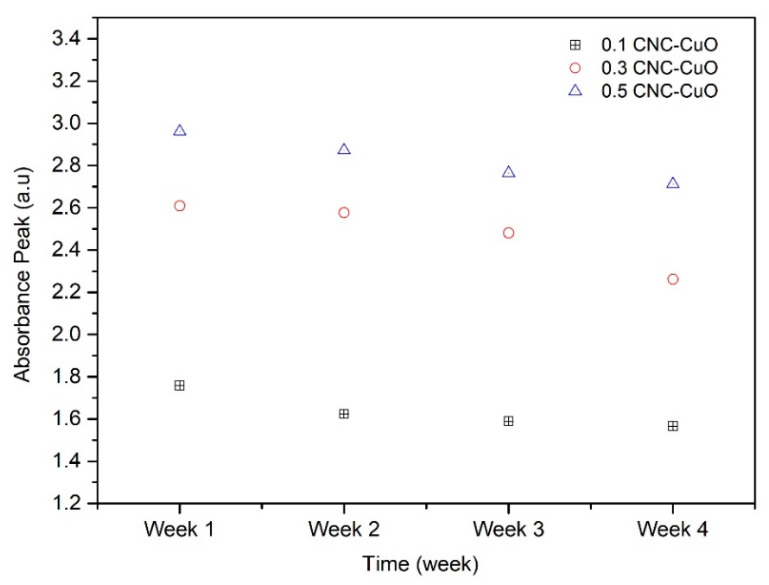
Value of absorbance peak every week.

**Figure 10:**
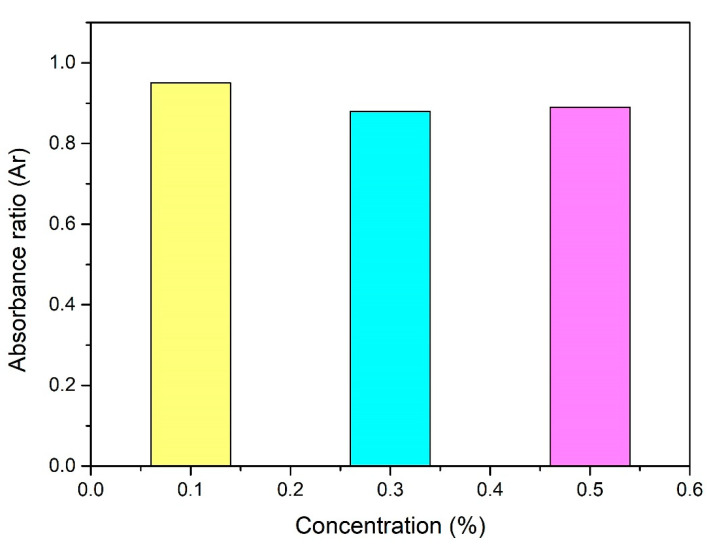
Absorbance ratio for all concentration.

**Figure 11:**
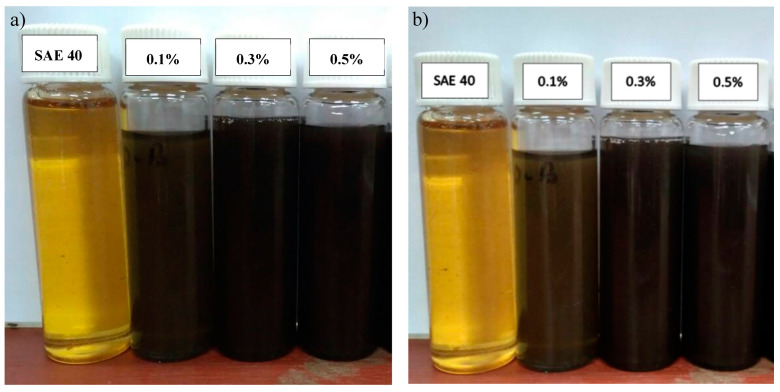
(**a**) Sedimentation at 0 week, (**b**) Sedimentation at 4 weeks.

The spectrum pattern at various volume concentrations of CNC-CuO lubricant is shown in Figure 8. It can be observed that the peak absorbance ranged from 0.1 to 0.5. The peak position was broadened due to the increase in CNC-CuO hybrids nanoparticle concentration. The absorbance observation was done after two months. Figure 8 shows the absorbance values at various volume concentrations of CNC-CuO nanoparticle with SAE 40. It can be observed that the peak absorbance for the 0.1 concentration occurs at a wavelength of 419 nm while for 0.3 and 0.5, it occurs at a wavelength of 415 nm. The higher peak of the absorbance level shows that the hybrid lubricant is stable. Figure 9 show the value of the absorbance peak at every week. It shows that the low concentration of nano lubricants sediment is faster due to rapid agglomeration [25]. The absorbance ratio indicates the ratio of the final absorbance at a specific sedimentation time towards the initial absorbance of the solution. The ideal absorbance ratio will be one or 100%, which demonstrates the excellent stability during the sedimentation period. According to Hajjar et al. [26] the closer the ratio is to one with the increase of the sedimentation times the more stable the sample is. Equation 1 determines the ratio of the final absorbance:

(1) $$Ar=\frac{A}{A_{o}}$$

where *A_r_* denotes the absorbance ratio, *A* denotes the final absorbance while *A_o_* denotes the initial absorbance. According to Figure 10, 0.1% shows the closest absorbance ratio to one; thus, 0.1% concentration shows the most stable nano lubricant, followed by 0.5% and the least stable nano lubricant is a 0.3% concentration. Figure 11 shows the sedimentation observation at the initial and at the fourth week. After the fourth week, the samples were found to be mixed well with no settlement of nanoparticles at the bottom of the test tube. Therefore, CNC-CuO nano lubricant was observed to be in stable condition for up to one month or more.

### 3.3. Effect of CNC-CuO on the Lubricant Viscosity

**Figure 12:**
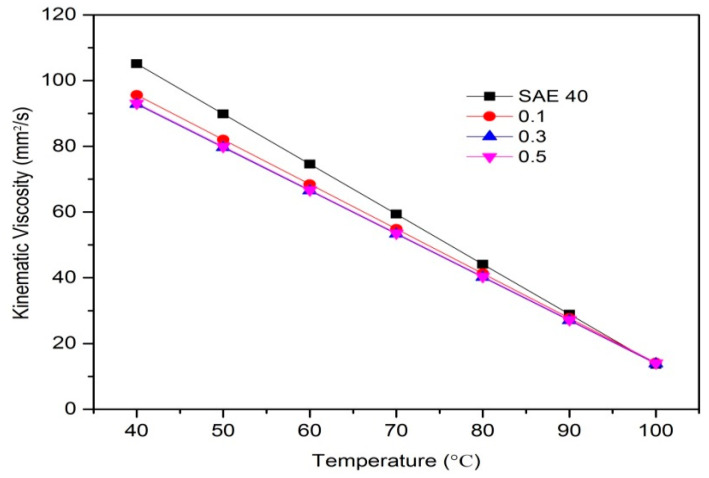
Kinematic viscosity of SAE 40 and different concentration of CNC-CuO nanolubricant.

**Figure 13:**
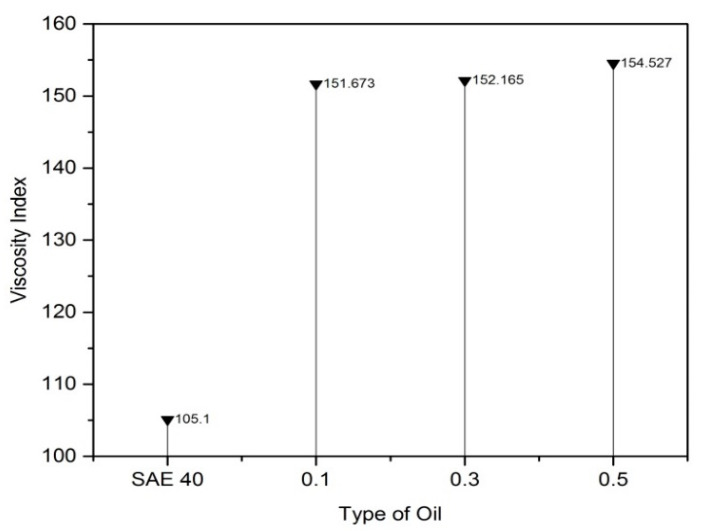
Viscosity index of SAE 40 and different concentrations.

According to Figure 12, SAE 40 shows higher kinematic viscosity values at 40 °C compared with another lubricant that contains CNC-CuO nanoparticles. CNC-CuO nanoparticle with a concentration of 0.1 shows a slightly higher kinematic viscosity while 0.3 and 0.5 do not show many differences between them. As the temperature of the viscosity approaches 100 °C, the nano lubricant kinematic viscosity value is close to the base oil SAE 40. Lubricant viscosity is the most important indication for lubricating testing because the viscosity of a lubricant is closely related to its ability to reduce friction in substantial body contacts. Generally, the least viscous lubricant is desirable [21]. This is because the systems oil pump works with less force to move a less viscous liquid. If the lubricant is too viscous, this will require a significant amount of energy to move while if it is too thin, the surfaces will come in contact and friction will increase [27]. In order to identify which lubricant exhibits better properties, the viscosity index (VI) was calculated and graphed, as shown in Figure 13. The lower the VI, the higher the change of viscosity of the oil with temperature. A higher VI was required to exhibit a better friction and wear [28]. According to Figure 12, as the concentration of CNC-CuO nanoparticle increases, the VI is higher, which can prove that CNC-CuO nanoparticle added with engine oil did improve the lubricity of the base oil regarding its viscosity by 44.3–47.12%.

### 3.4. Tribological Performance of CNC-CuO Nano Lubricant

#### 3.4.1. Friction and Wear Behaviour at High Low-Speed Load and High-Speed, Low Load

**Figure 14:**
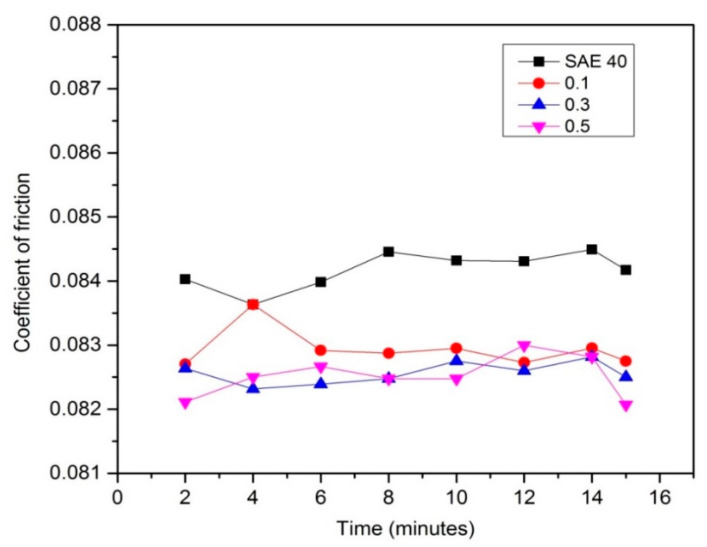
COF results for lubricant sample at low-speed high load.

**Figure 15:**
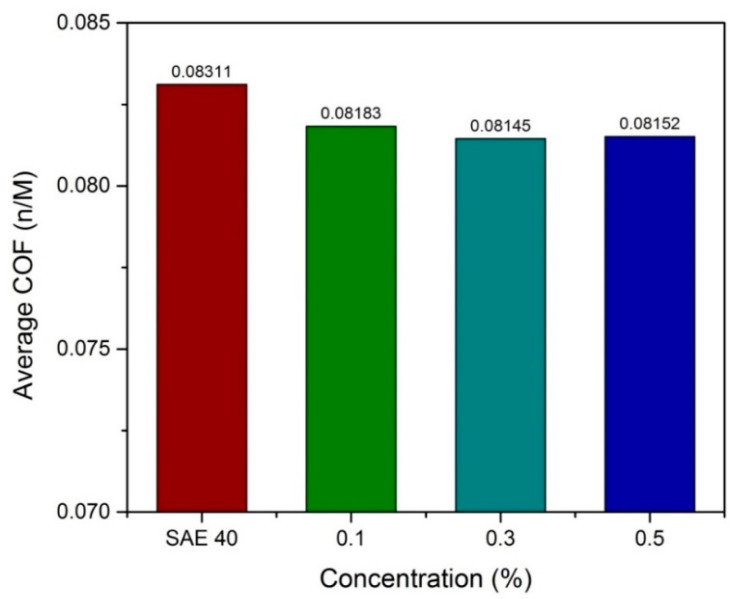
Average COF results at low-speed high load.

The coefficient of friction (COF) of base oil SAE 40 and nano lubricant at 0.1, 0.3 and 0.5 concentrations with low speed and high load is presented in Figure 14. At a low speed and high load, COF is the highest and at this state, the index lubrication value is known as the lambda value (λ) less than 1, which indicates the boundary lubrication regime at the Stribeck Curve [29]. As shown in Figure 14, SAE 40 clearly shows the highest friction compared to a lubricant that contents the CNC-CuO nanoparticle. From Figure 13 as well, the graph indicates the same pattern; at minute 2 until minute 8, COF starts to increase and slowly become constant at from minutes 8 to 12 and starts to drop at minutes 15 upwards. When the tribological tests at low speed and high load, the temperature of the friction region is 40 °C, which results in a decrease in the viscosities of the SAE 40 and CNC-CuO nanolubricants. The average COF result is shown in Figure 15.

**Figure 16:**
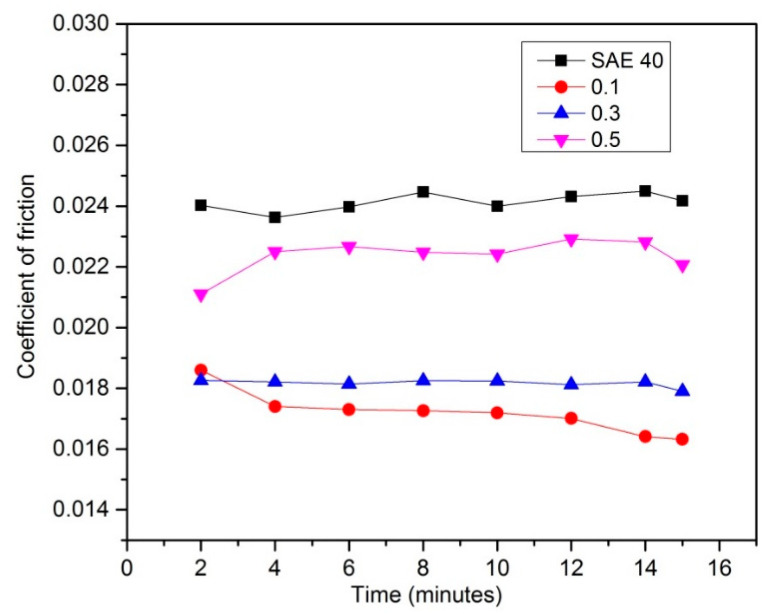
COF results for lubricant sample at high speed low load.

**Figure 17:**
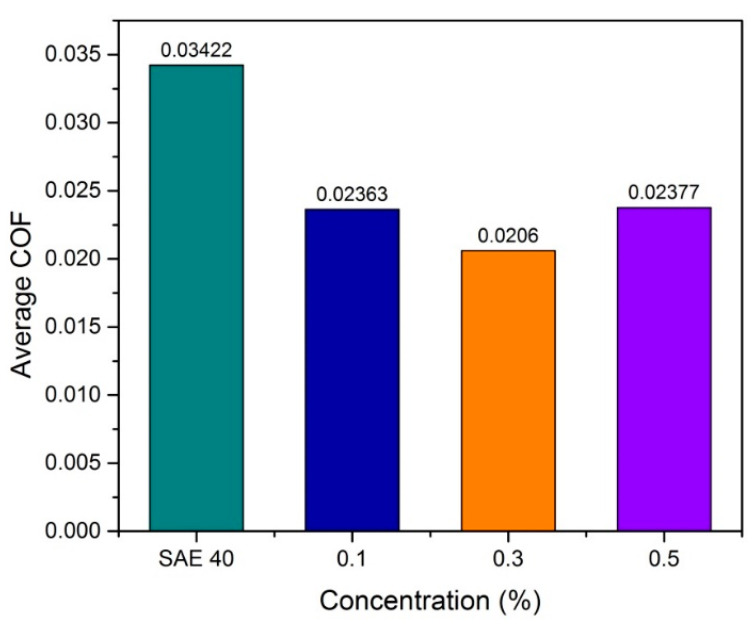
Average COF results at high speed low load.

**Figure 18:**
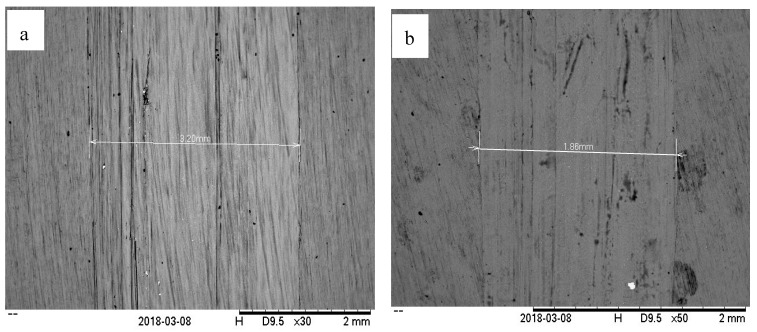
Wear quantity at (**a**) SAE 40 (**b**) 0.5.

At high speed and low load, the lambda value is always shows 1 until 3 and that indicates the mixed elastohydrodynamic lubrication, as proved by [29], and sometimes hydrodynamic lubrication, as proven by [30]. In this case, hydrodynamic lubrication is impossible since the time running the experiment is 30 min. Figure 16 shows the COF results versus time at high speed and low load. The result shows that SAE 40 produces the highest friction during sliding contact while as the concentration of CNC-CuO increases, the COF also increases. In the condition of high speed and low load, a lower friction makes the temperature of friction region decrease to 31 °C which makes the viscosity increase; thus, the increment of viscosity leads to low COF. Accordingly, the wear quantity of the sample specimen lubricated by SAE 40 is more significant than the sample specimen lubricated with 0.5 CNC-CuO. Figure 17 shows the average COF at results for high speed low load and it clearly shows that the improvement for COF at all concentrations. As shown in Figure 18, the wear track width lubricated by SAE 40 is 3.20 mm while for CNC-CuO, the nano lubricant is 1.86 mm. As can be seen in Figure 14 and Figure 16, only the break-in stage and the steady stage takes place since the running time is 15 min. The coefficient of friction increases during the break-in stage, which usually takes 6–12 min [31].

#### 3.4.2. Wear Mechanism

**Figure 19:**
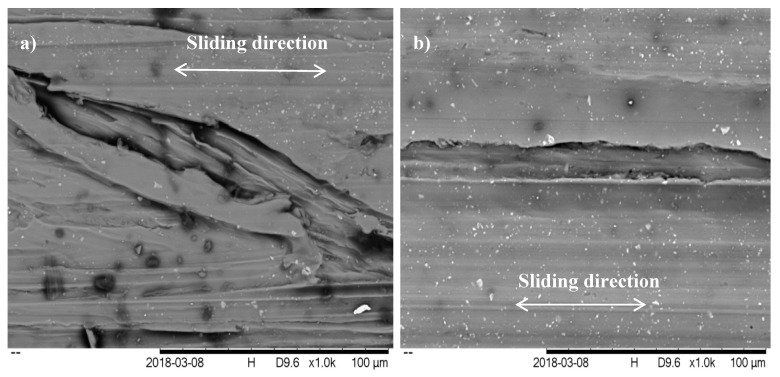
Wear morphologies at (**a**) SAE 40 [32] (figure reproduced with permission from Awang et al., published by Elsevier, 2019), (**b**) 0.5 CNC-CuO.

Morphologies tests were conducted at 100 N load and speed at 250 rev/min. This parameter was chosen for the observation because the highest friction occurs at low speed and high load [20] and compliance, as the result shows in Figure 14. Figure 19 compares the SEM results of the specimen surface with the base lubricant, SAE 40 and with 0.5% CNC-CuO nano lubricant use the same magnification (1000× magnification). It can be observed that some severe scuffing and exfoliations phenomenon occurred, as shown in Figure 18a, while light scuffing was found, as shown in Figure 18b. The extensive scratches happened due to micro-abrasive wear. This wear occurs due to tribofilm losing on the worn surface and becoming rough during the sliding. These wear results indicate that there is an improvement of scuffing and micro-abrasive wear while using CNC-CuO nano lubricant. It can also clearly be observed in Figure 19b that a CNC-CuO tribo-film was formed on the piston ring’s worn surface, thus, covering the significant scratches found in Figure 19a and leading to a smoother surface. Figure 19a was originally published in our previous work [32] and was inadvertently included in this manuscript as if it were original and new. The authors would like to clarify and correct this oversight. Figure 19a is identical to the image in reference [32], and shows SAE 40 engine oil at 100% concentration, representing the base image used in the tribology machine. As there was no observable difference in the appearance of the base SAE 40 engine oil across both studies, the same image is reused and is expected not to impact the scientific interpretation of the results. We acknowledge that the two studies differ in terms of the additive materials investigated.

**Figure 20:**
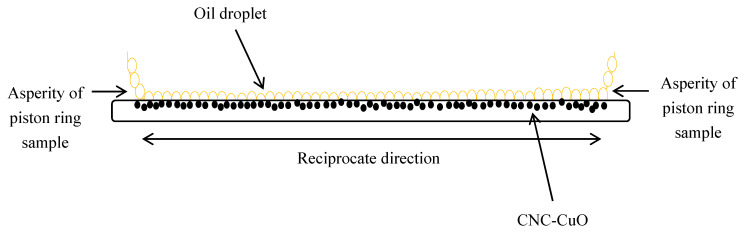
Front view of CNC-CuO based nano lubricant mechanism.

**Figure 21:**
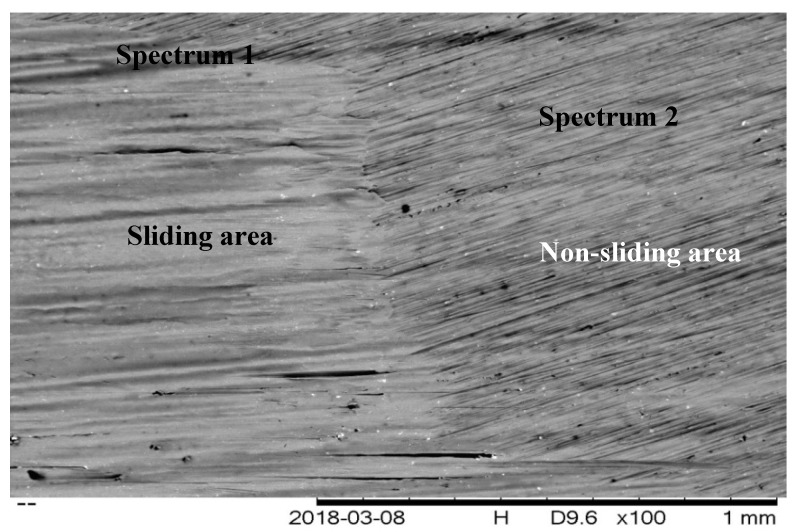
Sliding and non-sliding area of contact.

**Figure 22:**
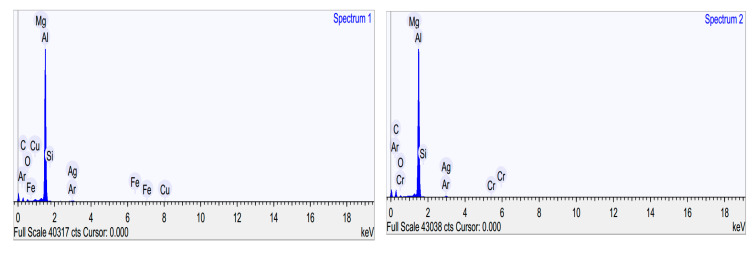
EDX spectrum of sliding and non-sliding contac.

This formation can also be confirmed through EDX studies, as shown in Table 1. The formation of CNC-CuO tribofilm helped to heal the cracks and the scratches of piston ring surface and cylinder liner including at the top dead centre, as shown in Figure 18a. It also proved the reduction of the average COF, as shown in Figure 14 and Figure 16. The schematic diagram of how CNC-CuO acts as a tribofilm is shown in Figure 20. Figure 21 shows the SEM pictures between the sliding and non-sliding areas. Cu and a higher percentage of O element were found in the sliding area, which indicates that the adhesive wear was found on the surface of the element, as shown in Table 2 and Figure 22. It also shows that there was a chemical reaction between the CNC-CuO and the metal surface, as Cu was the dominant element found in CuO nanoparticle and O element mainly from all organic nanoparticles, which may have contributed to a surface polishing effect.

## 4. Conclusions

Based on the thermo-physical and performance results, the characterization of hybrid CNC-CuO was achieved, from the stability of the nanolubricant and viscosity of lubricant together with the tribological behaviour of the nanolubricant. The conclusions from this work can be summarized as follows:

- The average CNC-CuO nanoparticle size is 82.6 nm. The peak absorbance for 0.1 concentration is 419 while for 0.3 and 0.5, is 415 nm.
- SAE 40 shows higher kinematic viscosity values at 40 °C than all another lubricants that contain CNC-CuO nanoparticles while between the concentration of the CNC-CuO nanoparticle, 0.1 shows a slightly higher kinematic viscosity while 0.3 and 0.5 do not show many differences. As the temperature of the viscosity approaches 100 °C, the nano lubricant kinematic viscosity value was close to the base oil (SAE 40). As for VI, as the concentration increases, VI increases.
- At low speed and high load, SAE 40 clearly shows the highest friction compared to a lubricant that contains CNC-CuO nanoparticle. At the initial stage, 0.1, 0.3 and 0.5 show almost the same COF while SAE 40 shows the highest friction. At low speed and high load, the temperature of the friction region is 40 °C, which results in a decrease in the viscosities of the SAE 40 and CNC-CuO nanolubricants. The average COF result also shows that SAE 40 is the highest, while at different concentrations of CNC-CuO, it did not show many differences. At high speed and low load, the result clearly shows that SAE 40 produces the highest friction during sliding contact while as the concentration of CNC-CuO increases, the COF also increases.
- The extensive scratches happened due to micro-abrasive wear. This wear occurs due to tribofilm losing on the worn surface and becoming rough during the sliding. These wear results indicate that there is an improvement of scuffing and micro-abrasive wear while using CNC-CuO nano lubricant. The wear is reduced with nanolubricant.

## Figures and Tables

**Table 1:** Dimensions of CNC.

Parameter	Value (nm)	Test Method
Crystal length	100–150	TEM
Crystal diameter	9–14	TEM
Hydrodynamic diameter	150	DLS

**Table 2:** Percentage of the element according to EDX spectrum.

Elements	Spectrum 1 (CNC-CuO)		Spectrum 2 (Non-Sliding Area)	
	Weight (%)	Atomic (%)	Weight (%)	Atomic (%)
Carbon	29.827	48.160	41.662	60.978
Oxygen	5.635	6.831	3.215	3.533
Magnesium	0.752	0.600	0.666	0.482
Aluminum	59.997	43.123	52.922	34.481
Silicon	0.421	0.290	0.318	0.199
Argon	0.258	0.125	0.370	0.163
Iron	0.912	0.317	-	-
Copper	1.263	0.386	-	-
Silver	0.936	0.168	0.691	0.113

## Abbreviations

CNC	Cellulose Nanocrystal
MWCNT	Multi Wall Carbon Nanotube
TEM	Transmission Emission Microscope
FESEM	Field Emission Scanning Microscope
EDX	Energy Dispersive Xray
COF	Coefficient of Friction
VI	Viscosity Index
AlO_2_	Aluminum Oxide
TiO_2_	Titanium Oxide
EG	Ethanol Glycol
CuO	Copper (II) Oxide
MgO	Magnesium Oxide
