# Correction: Hisham et al. Hybrid Nanocellulose-Copper (II) Oxide as Engine Oil Additives for Tribological Behavior Improvement. *Molecules* 2020, *25*, 2975

**DOI:** 10.3390/molecules30173527

**Published:** 2025-08-29

**Authors:** Sakinah Hisham, Kumaran Kadirgama, Hussein A. Mohammed, Amit Kumar, Devarajan Ramasamy, Mahendran Samykano, Saidur Rahman

**Affiliations:** 1 Faculty of Engineering Technology Mechanical and Automotive, Universiti Malaysia Pahang, Pekan 26600, Pahang, Malaysia; 2 School of Engineering, Edith Cowan University, 270 Joondalup Drive, Joondalup, WA 6027, Australia; 3 College of Engineering, Mechanical Department, Universiti Malaysia Pahang, Pekan 26600, Pahang, Malaysia; 4 Research Center for Nano-Materials and Energy Technology (RCNMET), School of Science and Technology, Sunway University, Bandar Sunway, Petaling Jaya 47500, Selangor, Malaysia; 5 Department of Engineering, Lancaster University, Lancaster LA1 4YW, UK

## Main Text Update

The authors confirm that the same images used in Figure 19 were indeed taken from the same laboratory for two publications [1,2]. N.W. Awang and S. Hashem were working on the same project, with the same affiliation from the same university and laboratory. Both supervisors were also co-authors on both papers. The image in question shows SAE 40 engine oil at 100%, and during the experiment with the tribology machine, they took only one image to document the results. This image serves as the base image of SAE 40 engine oil in the tribology machine. Since there is no significant difference in the appearance of the base SAE 40 engine oil, this is the main reason the same image was used in both papers. The projects differ in terms of the additive materials being studied. The authors confirm that there was no manipulation or use of images from other researchers.

To correct this error, the end of the first paragraph of Section 3.4.2. in the original publication [1] has been updated with the following paragraph:

Figure 19a was originally published in our previous work [32] and was inadvertently included in this manuscript as if it were original and new. The authors would like to clarify and correct this oversight. Figure 19a is identical to the image in reference [32], and shows SAE 40 engine oil at 100% concentration, representing the base image used in the tribology machine. As there was no observable difference in the appearance of the base SAE 40 engine oil across both studies, the same image is reused and is expected not to impact the scientific interpretation of the results. We acknowledge that the two studies differ in terms of the additive materials investigated.

## Correction in Figure Legend

The correct copyright permissions were obtained and provided to the Editorial Office for Figure 19, and the legend of Figure 19 in the original publication has been updated to the following:

**Figure 19.** Wear morphologies at (**a**) SAE 40 [32] (figure reproduced with permission from Awang et al., published by Elsevier, 2019), (**b**) 0.5 CNC-CuO.

## Added Citation

The following citation was added as reference 32 in the original publication.

32. Awang, N.W.; Ramasamy, D.; Kadirgama, K.; Najafi, G.; Che Sidik, N.A. Study on friction and wear of Cellulose Nanocrystal (CNC) nanoparticle as lubricating additive in engine oil. *Int. J. Heat Mass Transf.* **2019**, *131*, 1196–1204. https://doi.org/10.1016/j.ijheatmasstransfer.2018.11.128.

The authors state that the scientific conclusions are unaffected. This correction was approved by the Academic Editor. The original publication has also been updated.

## References

[1] Hisham S., Kadirgama K., Mohammed H.A., Kumar A., Ramasamy D., Samykano M., Rahman S. (2020). Hybrid Nanocellulose-Copper (II) Oxide as Engine Oil Additives for Tribological Behavior Improvement. Molecules.

[2] Awang N.W., Ramasamy D., Kadirgama K., Najafi G., Che Sidik N.A. (2019). Study on friction and wear of Cellulose Nanocrystal (CNC) nanoparticle as lubricating additive in engine oil. Int. J. Heat Mass Transf..

